# Cadm1-Expressing Synapses on Purkinje Cell Dendrites Are Involved in Mouse Ultrasonic Vocalization Activity

**DOI:** 10.1371/journal.pone.0030151

**Published:** 2012-01-17

**Authors:** Eriko Fujita, Yuko Tanabe, Beat A. Imhof, Mariko Y. Momoi, Takashi Momoi

**Affiliations:** 1 Center for Medical Science, International University of Health and Welfare, Kitakanemaru, Ohtawara, Tochigi, Japan; 2 Department of Pediatrics, Jichi Medical University, Yakushiji, Shimotsukeshi, Tochigi, Japan; 3 Department of Pathology and Immunology, Centre Médical Universitaire, University of Geneva, Geneva, Switzerland; Virginia Commonwealth University Medical Center, United States of America

## Abstract

*Foxp2*(R552H) knock-in (KI) mouse pups with a mutation related to human speech–language disorders exhibit poor development of cerebellar Purkinje cells and impaired ultrasonic vocalization (USV), a communication tool for mother-offspring interactions. Thus, human speech and mouse USV appear to have a Foxp2-mediated common molecular basis in the cerebellum. Mutations in the gene encoding the synaptic adhesion molecule CADM1 (RA175/Necl2/SynCAM1/Cadm1) have been identified in people with autism spectrum disorder (ASD) who have impaired speech and language. In the present study, we show that both *Cadm1*-deficient knockout (KO) pups and *Foxp2*(R552H) KI pups exhibit impaired USV and smaller cerebellums. Cadm1 was preferentially localized to the apical–distal portion of the dendritic arbor of Purkinje cells in the molecular layer of wild-type pups, and VGluT1 level decreased in the cerebellum of *Cadm1* KO mice. In addition, we detected reduced immunoreactivity of Cadm1 and VGluT1 on the poorly developed dendritic arbor of Purkinje cells in the *Foxp2*(R552H) KI pups. However, Cadm1 mRNA expression was not altered in the *Foxp2*(R552H) KI pups. These results suggest that although the Foxp2 transcription factor does not target Cadm1, Cadm1 at the synapses of Purkinje cells and parallel fibers is necessary for USV function. The loss of Cadm1-expressing synapses on the dendrites of Purkinje cells may be associated with the USV impairment that *Cadm1* KO and *Foxp2*(R552H) KI mice exhibit.

## Introduction

Cadm1 (also known as RA175, Necl2, and SynCAM1), a member of the immunoglobulin superfamily (IgSF), localizes to both sides of the synaptic cleft and functions as a synaptic cell–cell adhesion molecule. Cadm1 induces functional synapses [1]. The extracellular domain of Cadm1 mediates calcium-independent, homophilic *trans* interactions [1], [2], and its cytoplasmic tail has a band 4.1 region and a PSD95/Dlg/ZO-1 (PDZ)–binding motif [2]. At the pre-synapse, Cadm1 associates with calmodulin associated serine/threonine kinase (CASK) via a single PDZ domain [1].

Mutations in genes encoding synaptic adhesion proteins, including *neuroligin* (*NLGN*) 3 and 4, *contactin-associated protein-like 2* (*CNTNAP2*, *Caspr2*), and *CADM1*, are associated with autism spectrum disorder (ASD) [3]–[5]; the CADM1 mutations H246N and Y251S specifically have been found in people diagnosed with ASD who had impaired social interactions and communication, including speech and language impairments [5]. Mutations in CADM1 increase its susceptibility to processing errors and the accumulation of CADM1 peptide fragments in the endoplasmic reticulum [5], [6]; they also reduce CADM1 affinity in cell adhesion and lead to synaptic defects in neuron cultures [6]. *Cadm1* knockout (KO) mice [7] exhibit abnormal social and emotional behaviors that share similarities with some behaviors associated with ASD [8]. These findings suggest that CADM1 loss of function may be linked to ASD.

Speech–language impairment is one of the most prominent symptoms in some types of ASD. Impaired speech–language communication frequently also occurs as a phenotype of people with mutations in the adhesion molecule gene *CNTNAP2* [4]. A previous study found an R553H mutation in human *FOXP2* in patients with speech–language disorders [9]. Normal FOXP2 associates with a corepressor and acts as a transcriptional repressor [10]; however, mutated FOXP2 (R553H) lacks DNA-binding activity [11]. Infant mice emit and use ultrasonic vocalizations (USVs) as an essential communication tool for mother–offspring interactions [12]. *Foxp2* KO mice and knock-in (KI) mice for *Foxp2* (R552H), which corresponds to the human *FOXP2* (R553H) mutation, exhibit severe USV impairments, suggesting human speech and mouse USVs may have a common molecular basis in the brain [13], [14]. *Foxp2*(R552H) KI pups with USV impairment show poor development of Purkinje cells in the cerebellum [13], and the number of synapses on the dendrites of Purkinje cells is decreased in the these pups.

Of interest, cerebellar abnormalities, including Purkinje cell loss, have been found in autopsy samples from ASD patients [15]. We have observed that *Cadm1* KO mice have smaller cerebellums. Furthermore, Cadm1 mRNA is expressed not only in various regions of the cerebrum but also in the developing cerebellum [16]. Cadm1 is predominantly localized to the thalamus cortical afferent pathway in the cerebrum [17]; however, little is known about Cadm1 expression at synapses in the cerebellum.

In the present study, we examined USV of *Cadm1* KO mice, Cadm1 localization in the cerebellum, and the relationship between loss of Cadm1 at the synapses and impaired USV in *Cadm1* KO and *Foxp2*(R552H) KI pups.

## Results

**Figure 1:**
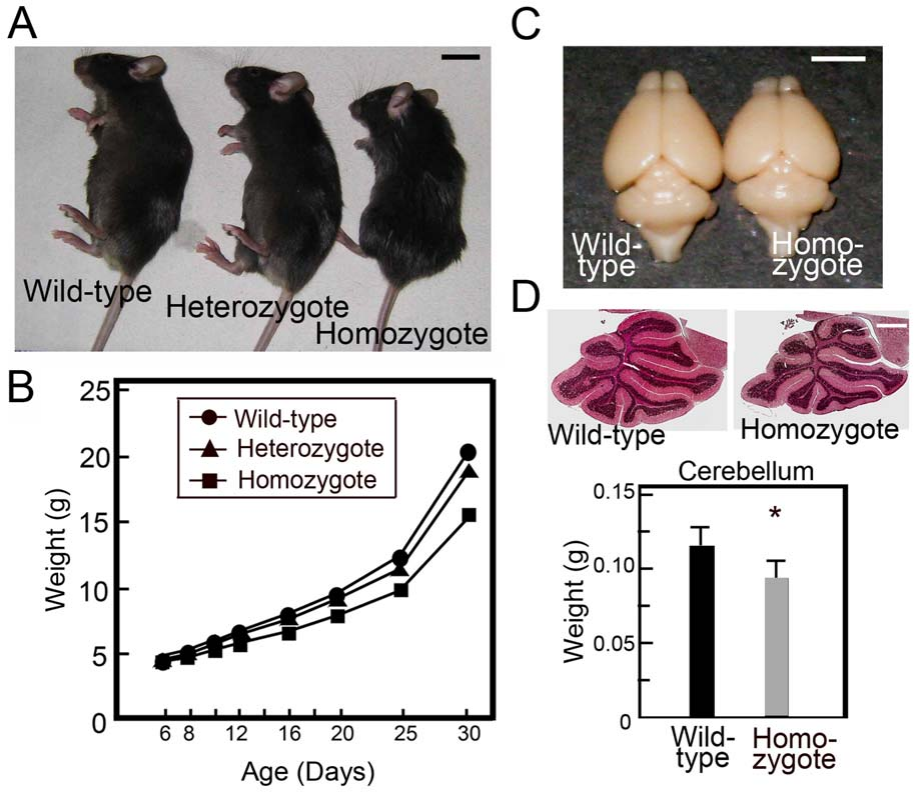
Abnormal cerebellum development of *Cadm1* KO. (A) Wild-type, heterozygote, and homozygous *Cadm1* KO mice. (P50) (B) The difference in mean weight between homozygous *Cadm1* KO mice and their wild-type littermates (five each) was significant at P10 and increased over the next 20 days (A, B); at P30, the mean weight of the homozygous *Cadm1* KO mice was 20–25% less than that of the wild-type mice. In addition, the brains of homozygous *Cadm1* KO mice were smaller (C, n = 22), and the cerebellums of homozygous *Cadm1* KO mice had an approximately 20% reduction in size and weight (D, n = 10). Bars in the graph indicate mean±standard error (SEM). Student's *t*-test (*p<0.05). Bars in the pictures indicate 1 cm (A), 5 mm (C), and 0.75 mm (D), respectively.

We established a strain of *Cadm1* KO (C57BL/6J) mice (*Cadm1* KO mice) by mating heterozygous *Cadm1* KO (129Sv) mice [7] with C57BL/6J for more than 10 generations. The homozygous *Cadm1* KO mice (postnatal day [P] 50) were smaller than their wild-type counterparts (Figure 1A). At P10, we detected a significant difference in mean body weight between homozygous *Cadm1* KO mice and their wild-type littermates, a difference that increased over the next 20 days. The mean body weight of the homozygous *Cadm1* KO mice was 20–25% less than that of the wild-type mice (Figure 1B). In addition, compared to the wild-type mice, the brains of homozygous *Cadm1* KO mice were smaller (Figure 1C). In particular, the cerebellum of homozygous *Cadm1* KO mice showed a reduction in size (Figure 1D, upper panel) and weight (Figure 1D, lower panel) of approximately 20%.

**Figure 2:**
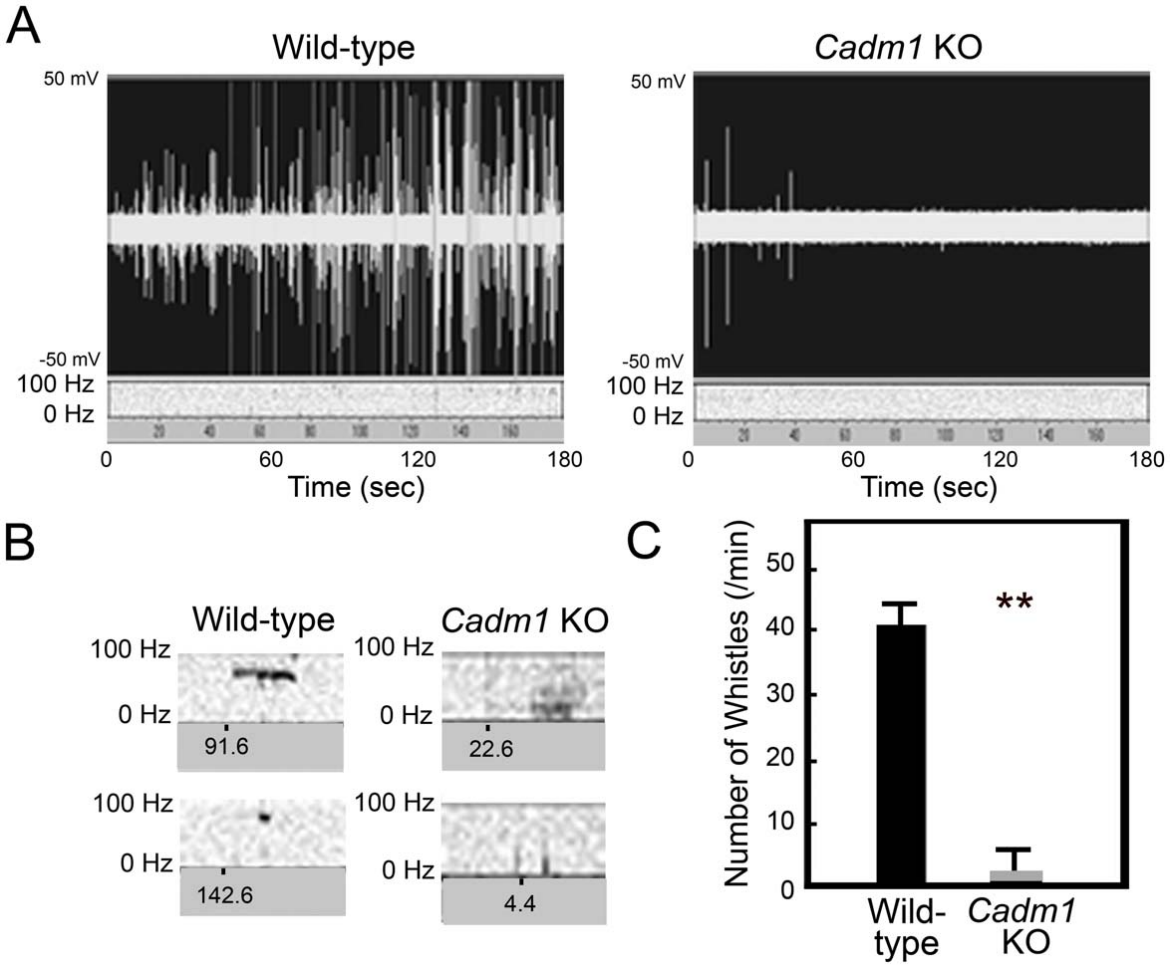
Analysis of ultrasonic vocalizations (USVs) of *Cadm1* KO mice (P8). (A) Real-time spectrography of the USVs by pups after separation from the dam. (B) Major vocalization patterns of *Cadm1* KO and wild-type pups. Wild-type vocalization was mainly whistle-type USVs, but *Cadm1* KO mice exhibited only a small number of click-type vocalizations. (C) The number of whistle-type USVs per min by pups. Vocalizations were recorded for 3 min. Experiments were done three times for 5 pups in each group, and an example of typical results is shown. Values are mean±standard error (SEM). Student's *t*-test (**p<0.01).

We next investigated the pups' USV because we previously found poor development of Purkinje cells in *Foxp2*(R552H) KI mice with impaired USV [13]. The *Cadm1* KO pups exhibited impaired USV upon separation from their mothers and litters, an effect similar to that which we recently observed in *Foxp2*(R552H) KI pups (Figure 2A) [13]. The *Cadm1* KO pups produced some click-type USVs but only low levels of whistle-type USVs, compared to the predominant whistle-type USVs among wild-type pups (Figure 2B, C).

**Figure 3:**
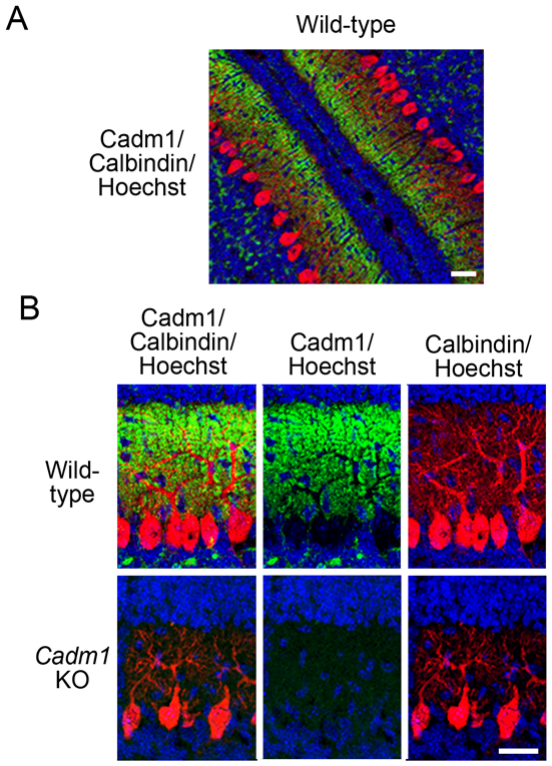
Distribution of Cadm1 in the cerebellum (P11). The Cadm1 intensity preferentially distributed in an apical–distal dendritic portion. Wild-type (A, B, upper panel) and *Cadm1* KO mice (B, lower panel). Green, Cadm1. Red, Calbindin. Blue, Hoechst. Bars, 30 µm.

The detection of these functional effects associated with Cadm1 deficiency led us to investigate more thoroughly the distribution pattern of Cadm1 in the cerebellum. In P11 wild-type pups, but not *Cadm1* KO pups, Cadm1 was detected in the dendritic arbor of Purkinje cells and some of the granular cells in the cerebellum (Figure 3A). Cadm1 preferentially localized to the apical–distal portion of the dendritic arbor (Figure 3B). The dendrite development of Purkinje cells in *Cadm1* KO mice appeared poor compared to that of wild-type mice (Figure 3B and Figure S1).

**Figure 4:**
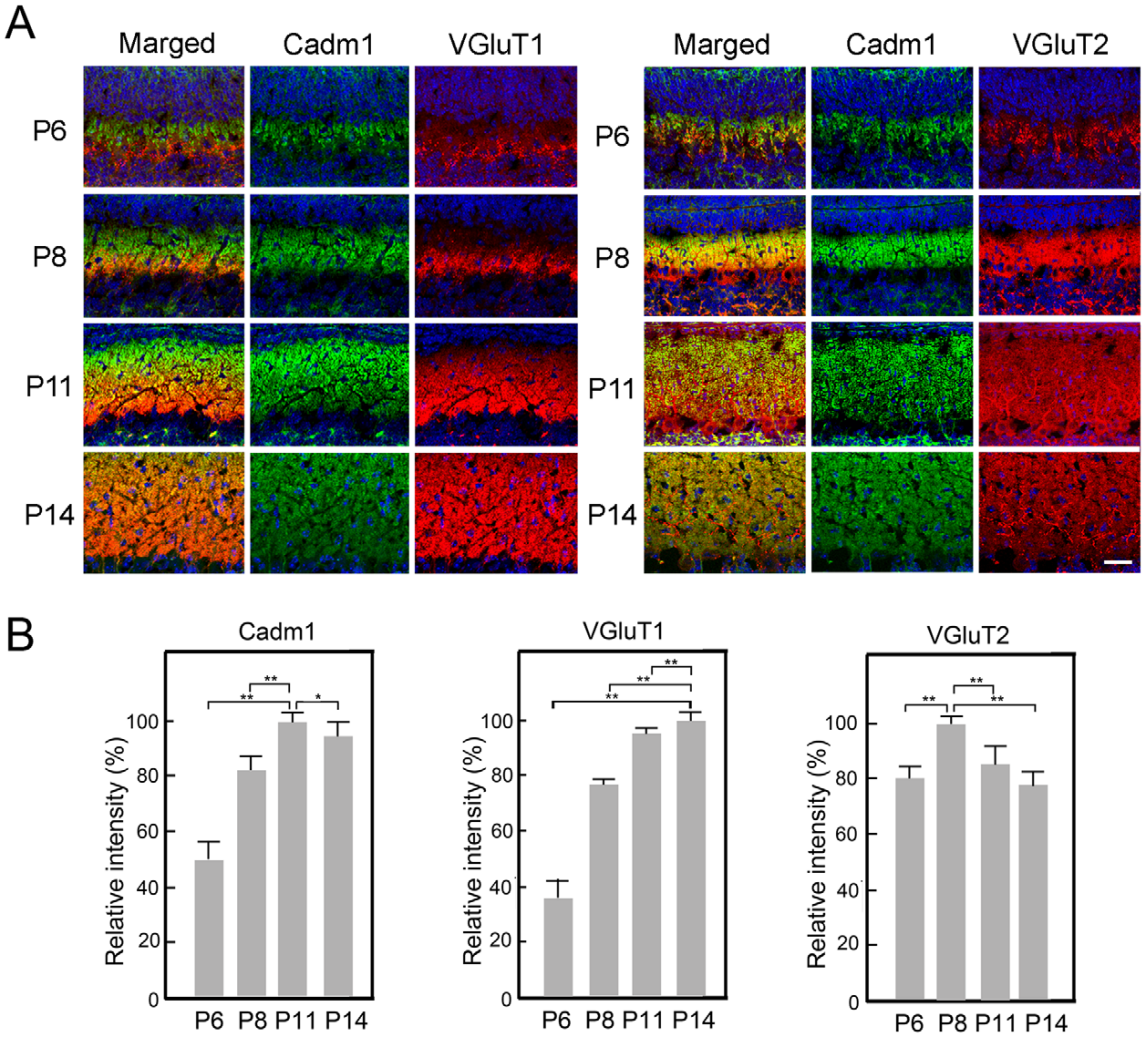
Developmental changes of Cadm1, VGluT1, and VGluT2 in wild-type pups. Alteration of the distribution of Cadm1, VGluT1, and VGluT2 was examined in the molecular layer of the developing cerebellum (P6–14). VGluT2 first appeared in the molecular layer in the early postnatal cerebellum (P6–8), in which Cadm1 co-localized with VGluT2, and then the level of VGluT2 decreased. VGluT1 increased in the later postnatal cerebellum (P11–14), in which Cadm1 co-localized with VGluT1. Green, Cadm1. Red, VGluT1 or VGluT2. Blue, Hoechst. Bar, 30 µm. Values are mean±standard error (SEM). Student's *t*-test (*p<0.05, **p<0.01). Pups: n = 3. Images: n = 8.

Purkinje cells receive two excitatory afferents, parallel fibers and climbing fibers, which can be distinguished based on the expression of VGluT1 and VGluT2 [18], [19]; climbing fibers express VGluT2 throughout development while parallel fibers shift from VGluT2 expression to VGluT1. The onset of VGluT2 expression in the individual parallel fiber terminals was clearly earlier than that of VGluT1 in the samples; in the early postnatal stages (P6–8), Cadm1 was mainly expressed in the molecular layer with the expression of VGluT2 (Figure 4A). During P6–11, Cadm1 expression intensity increased. At P11, VGluT2 intensity decreased, while VGluT1 intensity increased (Figure 4B). Thus, VGluT2 in parallel fibers expressing Cadm1 was replaced with VGluT1, which extended its expression from proximal regions to apical–distal regions in the molecular layer (Figure 4A). After this deep-to-superficial replacement, Cadm1 and VGluT1 immunoreactivity was detected throughout the molecular layer and appeared to co-localize at P14 (Figure 4A).

**Figure 5:**
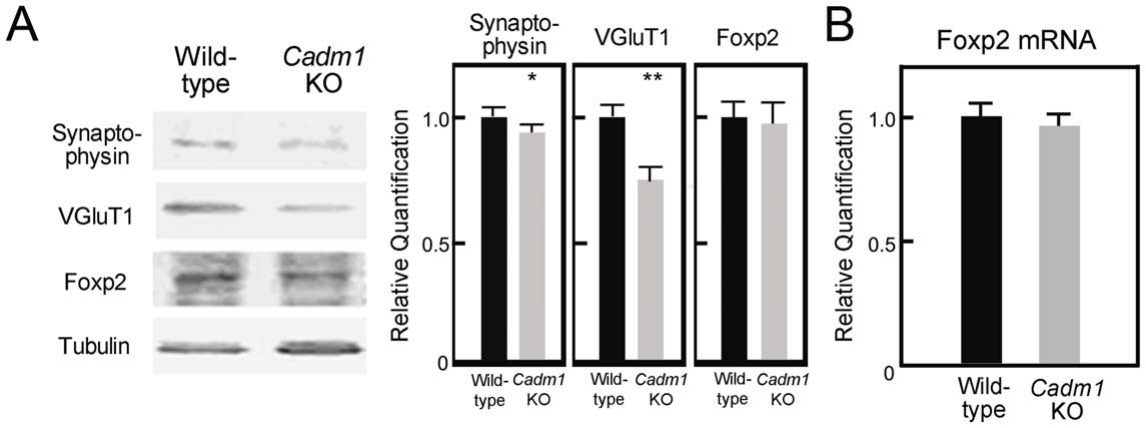
The influence of Cadm1 deficiency on the expression of synaptic proteins and Foxp2 in the cerebellum. (A) Immunoblot analysis of the influence of the deficiency in the cerebellums of *Cadm1* KO and wild-type pups (P10). An example of the typical immunoblotting results is shown. Values are mean±standard error (SEM). Student's *t*-test (*p<0.05, **p<0.01). Pups: n = 5. All experiments were performed three times. (B) RT-PCR analysis of the influence of the Cadm1 deficiency on the expression of *Foxp2* in the cerebellum of wild-type and *Cadm1* KO pups (P10). Values are mean±standard error (SEM). Pups: n = 5. All experiments were performed three times. A comparison showed no significant difference (Student's *t*-test; *p*<0.05).

We next examined the levels of Foxp2, Synaptophysin, and VGluT1 in the cerebellum of *Cadm1* KO mice (Figure 5A). VGluT1 levels were markedly decreased in the cerebellum of *Cadm1* KO compared to wild-type mice. Compared to VGluT1, the decrease in Synaptophysin was not marked, but it was significant; however, Foxp2 levels were unchanged. Real-time PCR analysis confirmed that there was no alteration in Foxp2 mRNA levels in the cerebellum of *Cadm1* KO compared to wild-type mice (Figure 5B).

Thus, Cadm1 deficiency did not appear to affect *Foxp2* expression and Foxp2-mediated development of Purkinje cell dendrites; however, it may have influenced synapse formation.

**Figure 6:**
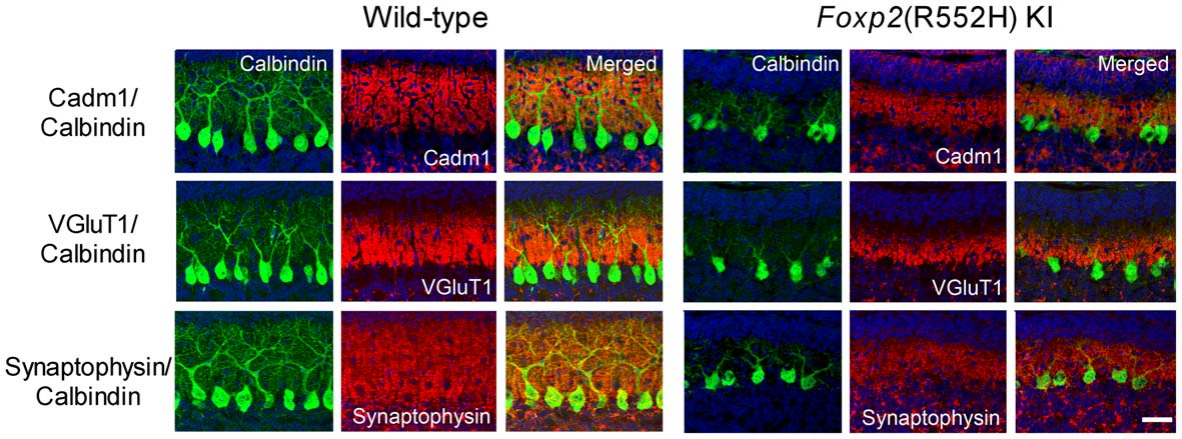
Altered distribution of Cadm1 in the molecular layer of wild-type and *Foxp2*(R552H) KI mice (P11). Cadm1 preferentially distributed in the apical–distal dendritic portion in the molecular layer. The immunoreactivity of Cadm1 as well as that of Synaptophysin and VGluT1, pre-synaptic markers, was decreased in the molecular layer of the *Foxp2*(R552H) KI mice. Green, Calbindin. Red, Cadm1, VGluT1, Synaptophysin. Blue, Hoechst. Bar, 30 µm.

We also examined the localization of Cadm1 in the cerebellum of *Foxp2*(R552H) KI mice and found that *Foxp2*(R552H) KI pups (P11) had poorly developed Purkinje cell dendrites with reduced immunoreactivity for Synaptophysin [13] (Figure 6). Overall, the immunoreactivity of Cadm1, as well as of VGluT1, was reduced on dendritic arbors in *Foxp2*(R552H) KI mice (Figure 6 and Figure S2), although Cadm1 mRNA levels were unchanged (Figure S3).

## Discussion

### Foxp2-mediated USV and Cadm1 activity in synapses in the cerebellum

Human speech and mouse USV have a common molecular basis in the brain, and *Foxp2*(R552H) KI mice exhibit abnormal cerebellar development and poor dendrite development [13]. In humans, some of the areas associated with speech and language skills are located in the frontal/superior cerebellar articulation control system and the parietal/inferior cerebellar phonological storage system [20], [21]. The cerebellar molecular systems control both human spoken language and mouse USVs and therefore share function in the two species.

In the present study, we found that *Cadm1* KO mice had smaller cerebellums, poor development of dendrites of Purkinje cells, and impaired USV (Figures 1, 2, 3 and S1), as observed in *Foxp2*(R552H) KI mouse pups. Cadm1 was preferentially localized to the apical–distal portion of the dendritic arbor of Purkinje cells in the molecular layer of wild-type pups (Figure 3), and the level of VGluT1 decreased in the cerebellum of *Cadm1* KO mice (Figure 5).

VGluT1/2-positive synapses have been detected in the brains of transgenic mice overexpressing *Cadm1* [22]. In the cerebellum, the two excitatory afferents of Purkinje cells are the parallel fibers and climbing fibers; climbing fiber terminals selectively express VGluT2 throughout the postnatal period, but parallel fiber terminals first express VGluT2 and then switch to VGluT1 [18], [19]. In the current work, Cadm1 was expressed in the granular cells and appeared to co-localize with VGluT1 at the pre-synapse (Figure 4). Both Cadm1 and VGluT1 immunoreactivity decreased in the Purkinje cells of *Foxp2*(R552H) KI pups (P11) with impaired USV (Figure 6), however. Of note, Cadm1 homophilically *trans* interacts at the synapse [1], [2]. In this study, at P11, in addition to VGluT1, Cadm1 partly co-localized with Synaptophysin, a pre-synaptic marker, and PSD-95, a post-synaptic marker, in the molecular layer (Figure S4). In a separate study, we found that Cadm1 also co-localized with GABBR2 on the dendrites of Purkinje cells during development (Fujita et al., submitted). Thus, Cadm1 may localize at the pre-synapse and post-synapse of the parallel fiber–Purkinje cells. The reduced immunoreactivity of Cadm1 on the dendrites of Purkinje cells in the *Foxp2*(R552H) KI mice could result from the decreased number of synapses. Foxp2 is essential for Purkinje cell development, while Cadm1 activity at parallel fiber–Purkinje cell synapses may be involved in mouse USV, and perhaps also in human spoken language. However, we note that loss of Cadm1 activity in other brain regions could also contribute to or even cause the vocalization phenotype, an important issue that future studies should address.

### *Cadm1* expression and Foxp2

The *CADM1* mutations H246N and Y251S have been identified in people with ASD who also had speech and language impairment [5]. In the current study, we found that *Cadm1* KO male mice (C57BL/6) had small cerebellums (Figure 1), impaired USV (Figure 2), and abnormal social and emotional behaviors, analogous to some behaviors associated with ASD [8].

ASD patients with mutations in the *CNTNAP2* gene also exhibit impaired speech and language [23]. A recent study showed that FOXP2 binds to the CAAATT motif in an intron of the human *CNTNAP2* gene, resulting in negative regulation of *CNTNAP2* expression; mutant FOXP2 (R553H) lacking DNA-binding activity resulted in increased *CNTNAP2* expression in *in vitro* experiments [11]. Human *CADM1* and mouse *Cadm1* have the same CAAATT binding motif for FOXP2 (accession no. NC_000011.9 for human *CADM1* and accession no. NC_000075.5 for mouse *Cadm1*). In contrast to CNTNAP2, we found here that Cadm1 mRNA levels were unchanged in the cerebellum of *Foxp2*(R552H) KI mice (Figure S1). Therefore, Foxp2 does not appear to regulate directly the expression of mouse *Cadm1* in the cerebellum. Thus, *Cadm1* and *CNTNAP2* exhibit different sensitivities to Foxp2 regulation, although they have the same CAAATT motif. This distinction may be attributable to different conditions in *in vitro* and *in vivo* experiments or to subtle variations in the binding motifs in the *Cadm1* and *CNTNAP2* genes; the nucleotide sequence of the repeated CAAATT motif, which is necessary for binding of dimerized Foxp2, may differ between the two genes.

In conclusion, *Cadm1* is not a target of the Foxp2 transcription factor, but Cadm1 activity at parallel fiber–Purkinje cell synapses may be necessary for USV function. Loss of Cadm1 activity at the synapse may be associated not only with USV impairment in mice but also with impaired speech and language communication skills in people with ASD.

## Materials and Methods

### Ethics statement

We followed the Fundamental Guidelines for Proper Conduct of Animal Experiments and Related Activities in Academic Research Institutions under the jurisdiction of the Ministry of Education, Culture, Sports, Science and Technology, and all of the protocols for animal handling and treatment were reviewed and approved by the Animal Care and Use Committee of Jichi University (approval numbers, H22-179, 10-179) and International University of Health and Welfare (approval numbers, D1008; 10118). Wild-type, *Cadm1* KO and *Foxp2*(R552H) KI mice [7], [13] (male mice) were used for the experiments.

### Ultrasonic vocalization

We mated *Cadm1* KO (129Sv) mice [7] with C57BL/6J strain mice for 10 generations and established a strain of *Cadm1* KO (C57BL/6J) mice. USVs of five *Cadm1* KO and five wild-type pups (P8) were assayed as described previously [13]. Briefly, each pup was separated from its mother and littermates, one at a time, placed in a shallow beaker in a soundproof chamber, and then positioned below a microphone connected to the UltraSound Gate 116 detector set (Avisoft Bioacoustics) to detect USVs of 40–100 kHz. Analysis began after the pup had been habituated to the chamber for 60 s. Sounds were recorded for 3 min.

### Quantitative real-time PCR

Total RNA was prepared from a combined five pieces of cerebellum of wild-type and *Cadm1* KO and *Foxp2*(R552H) KI male mice (P10), respectively, using the RNeasy mini kit (Qiagen) according to the manufacturer's specifications. Complementary DNAs were synthesized from total RNA (1 µg) using reverse transcriptase (Invitrogen) as described previously [24]. Real-time PCR analysis was performed using the Applied Biosystems 7500 fast real-time PCR system (Applied Biosystems) with the TaqMan Gene Expression Assays (Applied Biosystems) based on published sequences for genes encoding the respective mouse Cadm1, Foxp2, and VIC-labeled mouse Gapd (VIC-labeled MGD probe; Applied Biosystems) as endogenous control. For each sample, the 20 µl total volume consisted of 10 µl TaqMan Fast Universal PCR Master Mix (2x; Applied Biosystems), 1 µl TaqMan Gene Expression Assays, and 5 µl of each first-strand cDNA sample. The real-time PCR fragments were amplified as follows: 1 cycle at 95°C for 20 s, 60 cycles at 95°C for 3 s, and 60°C for 30 s. Results were analyzed using student's *t-*tests (*p*<0.05 was considered statistically significant).

### Immunblot analysis

Five cerebellums each from wild-type and *Cadm1* KO mice, respectively, were combined and lysed in lysis buffer [50 mM Tris-HCl pH 8.0, 150 mM NaCl, 10% glycerol, 0.5% IGEPAL CA630, and protease inhibitors; complete mini (Roche Diagnostics)] at 4°C for 15 min, and then each extract was subjected to immunoblot analysis using mouse anti-Synaptophysin (Millipore), rabbit anti-VGluT1 (Synaptic Systems), rabbit anti-Foxp2 (Abcam), and mouse anti-Tubulin (Sigma). Immunoreactivity was visualized using alkaline phosphatase-conjugated anti-mouse or anti-rabbit IgG, Nitro blue tetrazolium, and 5-bromo-4-chloro-3-indolyl-1-phosphate (Roche Diagnostics). Data from three experiments were scanned and analyzed for quantification with Image J software (National Institutes of Health). Results compared with wild-type were analyzed using the student's *t-*test (*p*<0.05 was considered statistically significant).

### Immunostaining

Wild-type, *Cadm1* KO, and *Foxp2*(R552H) KI mice cerebellums were fixed in 4% paraformaldehyde in phosphate buffered saline at 4°C overnight. Frozen sections (10 µm thick) were cut on a cryostat and immunostained with chicken anti-SynCAM1 (Cadm1; MBL), mouse anti-Calbindin (Sigma), rabbit anti-Calbindin (Sigma), mouse anti-Synaptophysin, rabbit anti-VGluT1, or rabbit anti-VGluT2 (Synaptic Systems). Alexa Fluor 488– and Alexa Fluor 568-conjugated secondary antibodies against mouse, rabbit, and goat IgGs were purchased from Molecular Probes. Nuclei were detected by Hoechst 33342 (Molecular Probes). The reactivity was viewed using a Leica SP5 confocal microscope (Leica Microsystems). At least three animals per genotype were examined, and experiments were repeated three times. Quantification of staining intensities was done using LAS AF software (Leica Microsystems). The mean pixel value in the area of interest and in the same size area of the background was calculated. The background level was subtracted from the value found in the area of interest (in the molecular layer). Reported intensities were normalized to control, and the Student's *t*-test was performed for statistical analysis.

## Supporting Information

Figure S1 **Alteration of Purkinje cells in cerebellum of wild-type and** ***Foxp2*** **(R552H) knock-in (** ***Foxp2*** **KI) mice, wild-type, and** ***Cadm1*** **knockout (** ***Cadm1*** **KO) (P11).** The immunoreactivity was performed using mouse anti-Calbindin. Bar, 20 µm.
(TIF)

Figure S2 **Altered distribution of the Cadm1 of wild-type and** ***Foxp2*** **(R552H) KI mice (P11).** Values are mean± standard error (SEM). Student's *t*-test (**p<0.01). Pups: n = 3. Images: n = 10.
(TIF)

Figure S3 **RT-PCR analysis of the expression of** ***Cadm1*** **in the cerebellum of wild-type and** ***Foxp2*** **(R552H) KI mice (P10).** Values are mean±standard error (SEM). Pups: n = 5. All experiments were performed three times. A comparison showed no significant difference (Student's *t*-test; *p*<0.05).
(TIF)

Figure S4 **The immunoreactivity (p11) of Synaptophysin (pre-synaptic marker) and PSD-95 (post-synaptic marker).** Green, Cadm1. Red, Synaptophysin or PSD-95 (Cell Signaling Technology). Blue, Hoechst. Bar, 30 µm.
(TIF)
