# Supplementary figures and images for: Cadm1-Expressing Synapses on Purkinje Cell Dendrites Are Involved in Mouse Ultrasonic Vocalization Activity
Source: PLoS One. 2012 Jan 17;7(1):e30151. doi: 10.1371/journal.pone.0030151 (PMC3260241; doi:10.1371/journal.pone.0030151)

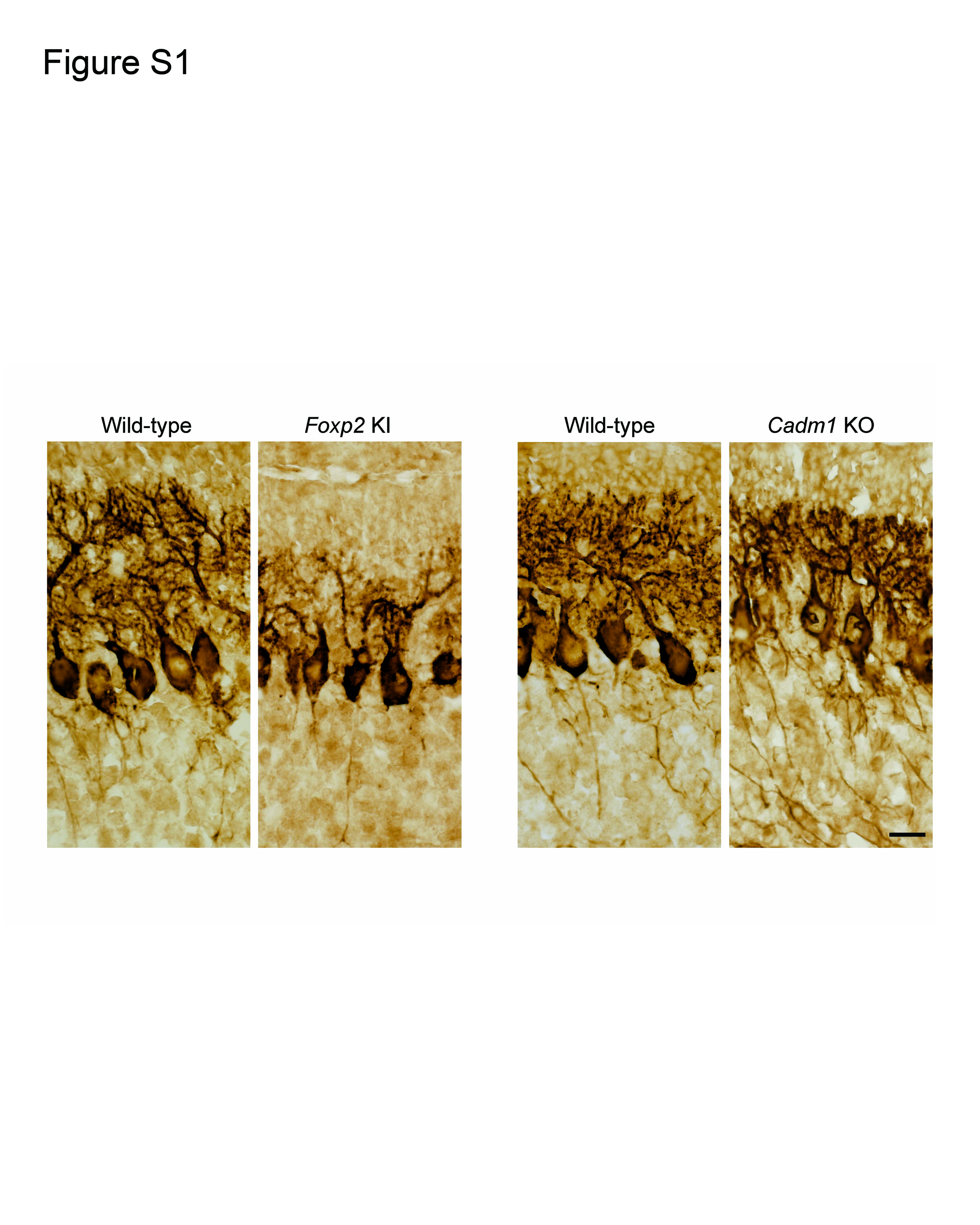

Supplement: Figure S1 — Alteration of Purkinje cells in cerebellum of wild-type and Foxp2 (R552H) knock-in ( Foxp2 KI) mice, wild-type, and Cadm1 knockout ( Cadm1 KO) (P11). The immunoreactivity was performed using mouse anti-Calbindin. Bar, 20 µm. (TIF) [file pone.0030151.s001.tif]

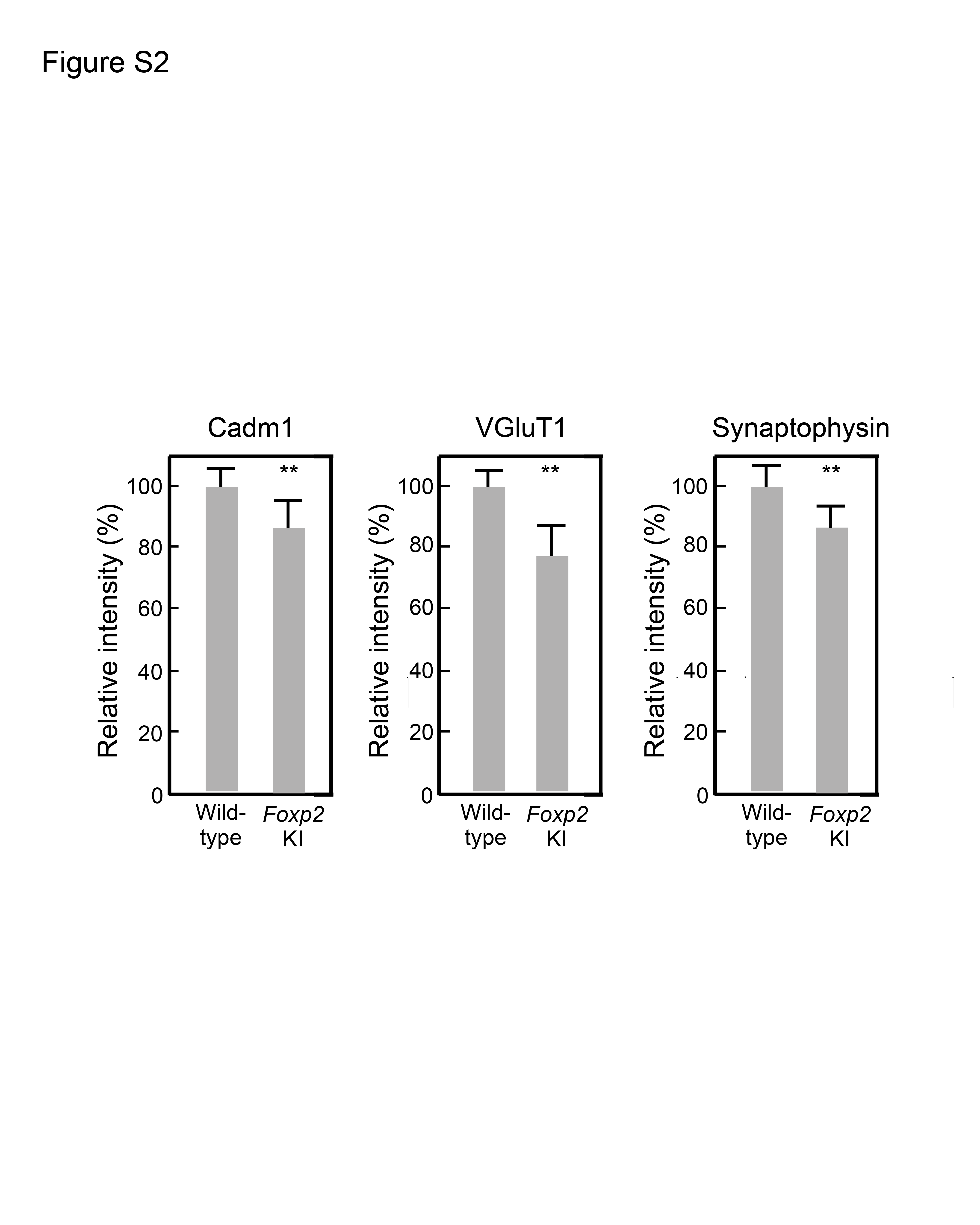

Supplement: Figure S2 — Altered distribution of the Cadm1 of wild-type and Foxp2 (R552H) KI mice (P11). Values are mean± standard error (SEM). Student's t-test (**p<0.01). Pups: n = 3. Images: n = 10. (TIF) [file pone.0030151.s002.tif]

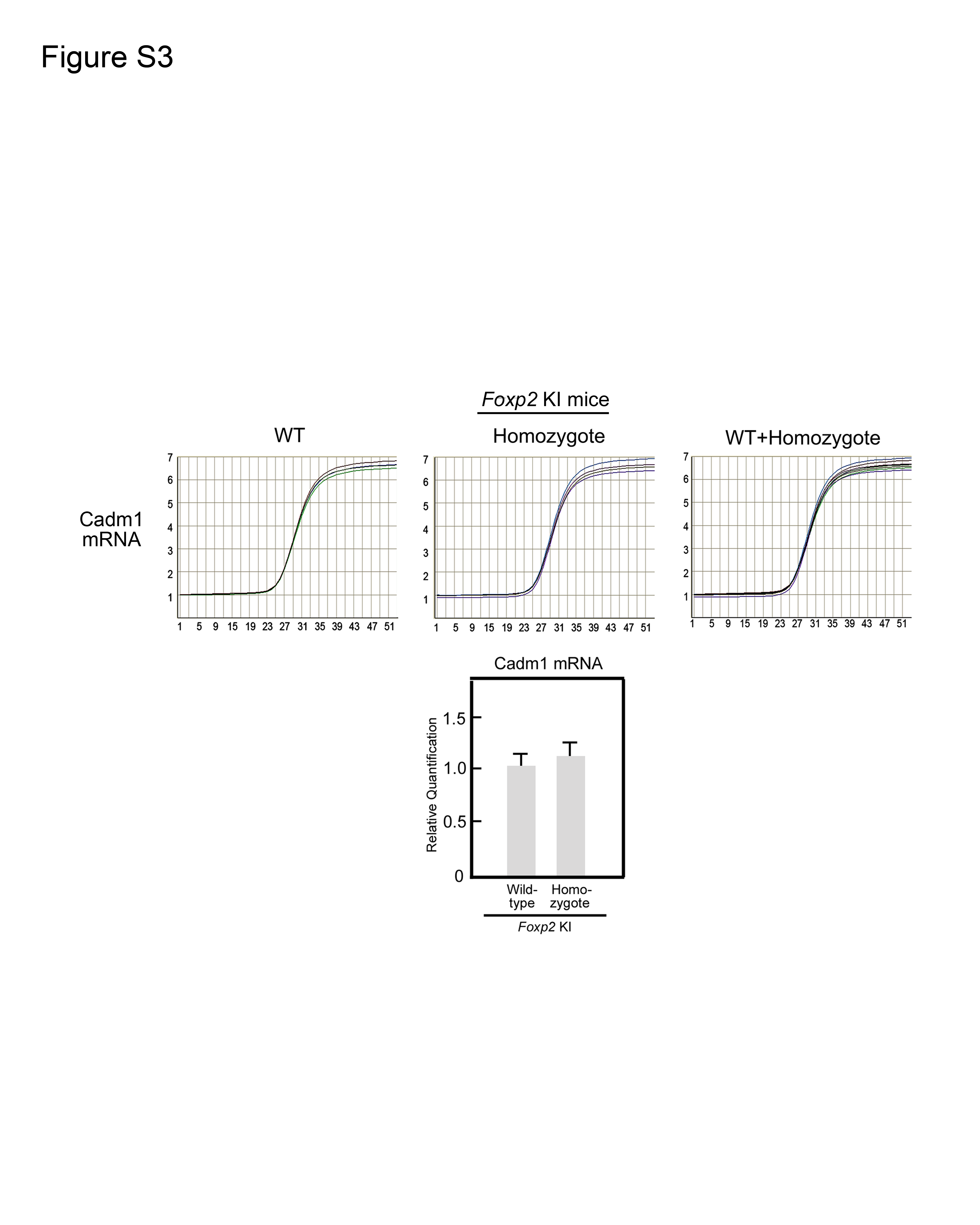

Supplement: Figure S3 — RT-PCR analysis of the expression of Cadm1 in the cerebellum of wild-type and Foxp2 (R552H) KI mice (P10). Values are mean±standard error (SEM). Pups: n = 5. All experiments were performed three times. A comparison showed no significant difference (Student's t-test; p<0.05). (TIF) [file pone.0030151.s003.tif]

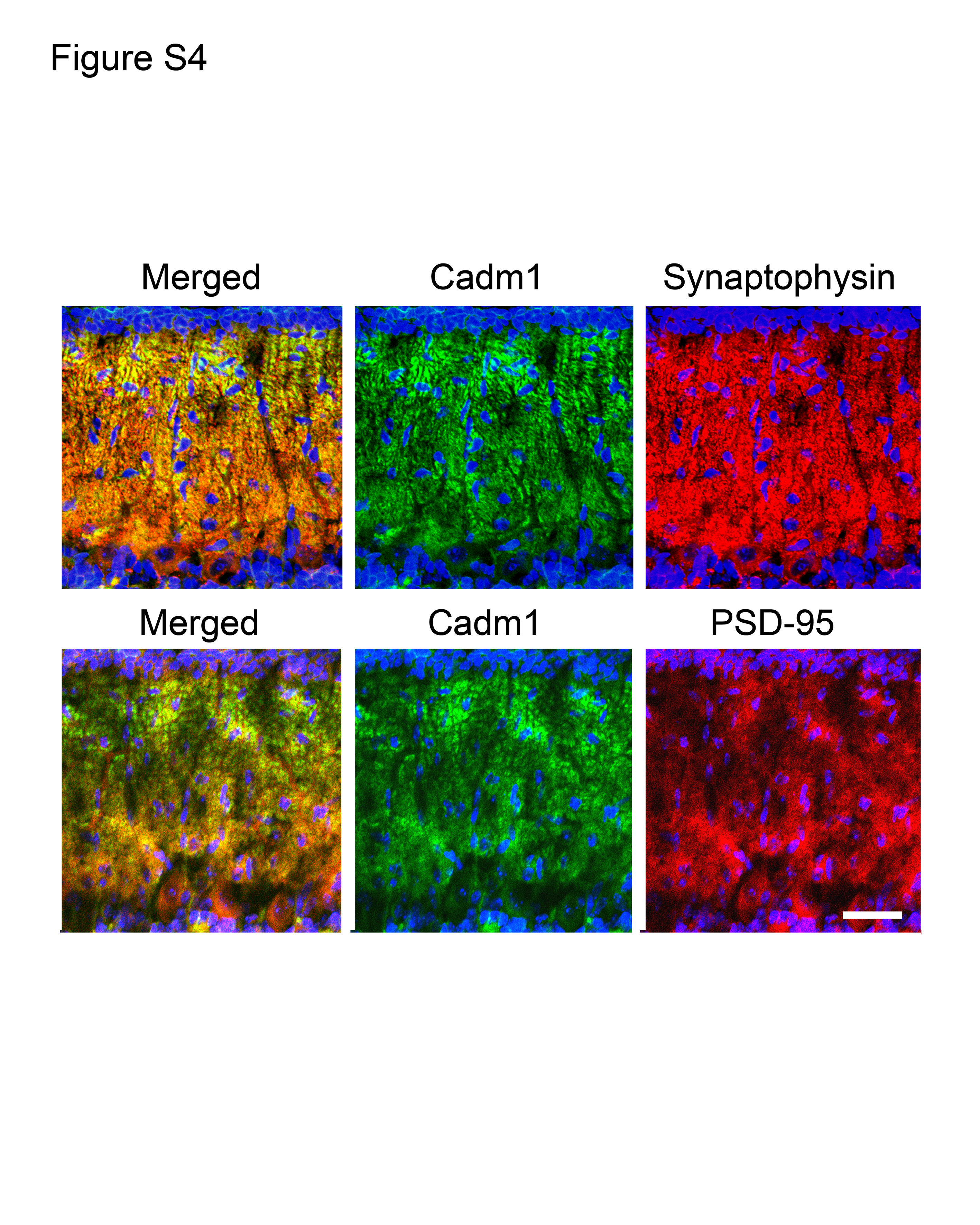

Supplement: Figure S4 — The immunoreactivity (p11) of Synaptophysin (pre-synaptic marker) and PSD-95 (post-synaptic marker). Green, Cadm1. Red, Synaptophysin or PSD-95 (Cell Signaling Technology). Blue, Hoechst. Bar, 30 µm. (TIF) [file pone.0030151.s004.tif]
